# Hesperidin promotes gastric motility in rats with functional dyspepsia by regulating Drp1-mediated ICC mitophagy

**DOI:** 10.3389/fphar.2022.945624

**Published:** 2022-08-12

**Authors:** Qingling Jia, Li Li, Xiangxiang Wang, Yujiao Wang, Kailin Jiang, Keming Yang, Jun Cong, Gan Cai, Jianghong Ling

**Affiliations:** Department of Gastroenterology, Shuguang Hospital, Shanghai University of Traditional Chinese Medicine, Shanghai, China

**Keywords:** hesperidin, functional dyspepsia, mitophagy, mdivi-1, Drp1

## Abstract

Hesperidin is one of the main active ingredients of *Citrus aurantium* *L.* (Rutaceae) and tangerine peel, which have anti-inflammatory and antioxidant effects. In previous study, we found that gastric motility disorder in functional dyspepsia (FD) rats accompanied by excessive autophagy/mitochondrial swelling and even vacuolization in the interstitial cells of cajal (ICC), but the exact mechanism has not yet been investigated. Therefore, we used different doses of hesperidin (50 mg/kg, 100 mg/kg, and 200 mg/kg) to intervene in FD rats, and found that medium doses of hesperidin (100 mg/kg) significantly increased gastric motility in FD rats. Subsequently, FD rats were randomly divided into control group, model group, mdivi-1 group, mdivi-1+hesperidin group and hesperidin group, and mitochondrial division inhibitor (mdivi-1) was injected intraperitoneally to further investigate whether hesperidin could regulate dynamin-related protein 1 (Drp1)-mediated mitophagy in ICC to improve mitochondrial damage. The results showed that compared with the model group, the serum malondialdehyde (MDA) level decreased and the superoxide dismutase (SOD) level increased in the mdivi-1 and hesperidin groups (*p* < 0.001). Transmission electron microscopy (TEM) observed that the mitochondrial nuclear membrane was intact in gastric tissues with a clear internal cristae pattern, and autophagy lysosomes were rare. The co-localization expression of microtubule associated protein 1 light chain 3 (LC3) and voltage dependent anion channel 1 (VDAC1), Drp1 and translocase of the outer mitochondrial membrane 20 (Tom20) was significantly decreased (*p* < 0.001), the protein expression of mitochondrial Drp1, Beclin1 and LC3 were significantly decreased (*p* < 0.001), the protein expression of mitochondrial P62 and ckit in gastric tissue were significantly increased (*p* < 0.05, *p* < 0.001). The above situation was improved more significantly by the synergistic intervention of mdivi-1 and hesperidin. Therefore, hesperidin can improve mitochondrial damage and promote gastric motility in FD rats by regulating Drp1-mediated ICC mitophagy.

## 1 Introduction

Functional dyspepsia (FD) is a common gastrointestinal disorder with a reported global prevalence of 5–11% in the community, which has caused a severe social and economic burden (Talley and Ford, 2015; Jin and Son, 2018; Kim et al., 2018). Currently, gastric motility disorders are considered to be one of the pathophysiological features of FD and are closely associated with dyspepsia symptoms (Asano et al., 2017). Gastric motility is mostly regulated by the autonomic and enteric nervous systems as well as by the interstitial cells of cajal (ICC) (Naganuma et al., 2017). As a pacemaker of gastrointestinal slow-wave activity, ICC plays an important role in intestinal motility by transmitting excitatory and inhibitory signals to affect gastrointestinal motility (Hagger et al., 1997). Ckit is a specific receptor for ICC in the gastrointestinal tract, and ICC is cell that specifically express ckit. Studies have shown that blockade of ICC development in BALB/c mice by the anti-ckit monoclonal antibody ACK2 leads to severe abnormalities in intestinal motility, reduces the number of ICC in the small intestine, and inhibits ICC proliferation and development (Torihashi et al., 1995; Streutker et al., 2007). In our previous study, we found that impaired gastric motility in FD rats was accompanied by excessive autophagy/mitochondrial swelling and even vacuolization of ICC (Tan et al., 2019).

Mitophagy is a form of cellular autophagy that occurs when mitochondria are depolarized and damaged by external stimuli such as oxidative damage, and the damaged mitochondria are wrapped in autophagosomes and fused with lysosomes, thus completing the degradation of the damaged mitochondria (Patergnani and Pinton, 2015; Springer and Macleod, 2016). Mitochondrial division inhibitor (mdivi-1) is a selective inhibitor of dynamin-related protein 1 (Drp1) that inhibits Drp1-mediated mitochondrial division by inhibiting GTPase activity (Duan et al., 2020). Mdivi-1 treatment has been shown to prevent lipopolysaccharide-induced mitophagy and reduce acute lung injury in rats (Luo et al., 2019). Activation of mitophagy via a Drp1-dependent pathway reduced apoptosis and improved renal ischaemia/reperfusion injury-induced renal dysfunction (Li et al., 2018). Furthermore, mdivi-1 prevents the initiation of mitophagy in diabetic cardiomyocytes (Mishra et al., 2014). However, no studies have yet shown how mitophagy plays a role in FD, and further studies are warranted.

Hesperidin, a flavonoid, is one of the main active components of traditional Chinese medicines *Citrus aurantium L.* (Rutaceae) and tangerine peel, and has anti-inflammatory, antioxidant, analgesic, and antihypertensive effects (Iranshahi et al., 2015; Parhiz et al., 2015). Studies have shown that hesperidin can effectively improve gastrointestinal function in rats. For example, hesperidin can improve colonic motility in loeramide-induced constipation rats through the serotonin 4R/cAMP signaling pathway (Wu et al., 2019). Hesperidin may play an anti-ulcer effect by preventing hemorrhagic injury of gastric mucosa through its antioxidant effect (Bigoniya and Singh, 2014). In addition, hesperidin can reduce oxidative stress and mitochondrial dysfunction by inhibiting DNA Methyltransferase 1-mediated silencing of miR-149, and ameliorate high glucose-induced insulin resistance (Tian et al., 2021). Although hesperidin is known to alleviate FD, its specific mechanism has been rarely studied. Therefore, in this study, we aimed to explore the mechanism of hesperidin through Drp1-mediated ICC mitophagy, improving mitochondrial damage and promoting gastric motility in FD rats.

## 2 Methods and materials

### 2.1 Animals

66 specific pathogen-free (SPF) SD rats, 5–6 weeks old, body weight (200 ± 20 g), purchased from Zhejiang Weitong Lihua Laboratory Animal Technology Co., Ltd., certificate number SCXK (Zhejiang) 2019-0001, housed in SPF-grade barrier environment at the Experimental Animal Center of Shanghai University of Traditional Chinese Medicine (TCM), with alternating light and dark cycles of 12/12 h, relative temperature (22 ± 2)°C and relative humidity (55 ± 2)%. This study was approved by the Animal Ethics Committee of Shanghai University of TCM (PZSHUTCM210108004).

### 2.2 Reagents

Hesperidin (Dalian Meilun Biotechnology Co., Ltd., MB6567, Dalian, China), Domperidone Tablets (Xian Janssen Pharmaceutical Ltd., KDJ3YSP, Xian, China), mdivi-1 (Shanghai Selleck, S7162, Shanghai, China), rabbit anti-Drp1 (Abcam, ab184247, Cambridge, UK), rabbit anti-ckit (Cell Signaling Technology, 3074, MA, United States), rabbit anti-Beclin1 (Cell Signaling Technology, 3738S), rabbit anti-P62 (Cell Signaling Technology, 23214S), rabbit anti-LC3 (Cell Signaling Technology, 4108S), rabbit anti-GAPDH (Cell Signaling Technology, 5174S), anti-rabbit IgG, HRP-linked antibody (Cell Signaling Technology, 7074P2), mouse anti-VDAC1 (Santa Cruz Biotechnology, Inc., sc-390996, CA, United States), mouse anti-Tom20 (Santa Cruz Biotechnology, Inc., sc-17764), Bovine Serum Albumin (BSA) (Shanghai Beyotime Biotechnology Co., Ltd., ST023, Shanghai, China), Alexa Fluor 488-labeled Goat Anti-Rabbit IgG (H + L) (Shanghai Beyotime Biotechnology Co., Ltd., A0423), Cy3-labeled Goat Anti-Rat IgG (H + L) (Shanghai Beyotime Biotechnology Co., Ltd., A0507), Antifade Mounting Medium with DAPI (Shanghai Beyotime Biotechnology Co., Ltd., P0131), SDS-PAGE Gel Quick Preparation Kit (Shanghai Beyotime Biotechnology Co., Ltd., P0012AC), Mitochondrial Extraction Kit (Beijing Solarbio Science & Technology Co., Ltd., SM0020, Beijing, China), EZ-Buffers H 10X TBST Buffer (Shanghai Sangon Biotech Co., Ltd., C520009, Shanghai, China), Reactive Oxygen Species (ROS) Assay Kit (Nanjing Jiancheng Bioengineering Institute, E004-1-1, Nanjing, China), Rat Motilin (MTL) ELISA Kit (Shanghai Biological Technology Co., Ltd. enzyme research, EK-R30889, Shanghai, China), Rat Gastrin (GAS) ELISA Kit (Shanghai Biological Technology Co., Ltd. enzyme research, EK-R30890), Rat Superoxide Dismutase (SOD) ELISA Kit (Shanghai Jianglai Biotechnology Co., Ltd., JL22893, Shanghai, China), Rat Malondialdehyde (MDA) ELISA Kit (Shanghai Jianglai Biotechnology Co., Ltd., JL13297).

### 2.3 Grouping, modelling and drug administration

After 1 w of adaptive feeding, 36 SD rats were randomly divided into control group (C), model group (M), hesperidin low-dose group (Hes + low), hesperidin medium-dose group (Hes + medium), hesperidin high-dose group (Hes + high), and Domperidone (Dompe) group, six rats in each group. All groups, except the control group, were modelled with reference to the modified tail-clamping stimulation method (Wu et al., 2020; Li et al., 2022) for 30 min each time, 2 times/d for 4 w. The dose of gavage was calculated according to the equivalent dose formula of 60 kg adult body weight and rats, and the model group was given saline gavage at 1.5 ml/100 g, the Hes + low group, Hes + medium group, Hes + high group and Dompe group were given 50 mg/kg, 100 mg/kg, 200 mg/kg and 4.5 mg/kg aqueous solution by gavage once in the morning and once in the evening at an interval of 12 h for 4 w. Subsequently, 30 SD rats were randomly divided into control group (C), model group (M), mdivi-1 group (mdivi-1), mdivi-1+hesperidin group (mdivi-1+Hes), hesperidin group (Hes), six rats in each group. Mdivi-1 was injected intraperitoneally once every other week at a dose of 25 mg/kg, and the gavage dose for the Hes group was 100 mg/kg.

### 2.4 Preparation of semi-solid paste

5 g of sodium carboxymethyl cellulose, 8 g of skim milk powder, 4 g of starch, and 4 g of sugar were dissolved in 125 ml of distilled water, and fully mixed to make 150 ml of nutritional semi-solid paste.

### 2.5 Specimen collection and processing

After the last administration, the rats were fasted for 12 h. The next day, the rats in each group were gavaged with the semi-solid paste, and 30 min later, the rats were anesthetized with 2.5% sodium pentobarbital (2.25 ml/kg, ip), dissected along the side of the greater curvature of the stomach, and blood was removed from the abdominal aorta by flushing with ice-cold saline, then blotted and spread on filter paper. The whole stomach was exposed, the cardia and pylorus were quickly ligated with surgical thread, the connective tissue on the surface of the stomach body was stripped, the mass of the whole stomach was weighed and recorded, the stomach was cut along the greater curvature, the stomach contents were rinsed with saline, the filter paper was blotted dry, the mass of the empty stomach was weighed and recorded, the tissue of the gastric sinus was taken, part of it was fixed in 5% glutaraldehyde and 4% paraformaldehyde respectively, part of it was extracted from the mitochondria according to the instructions of the mitochondrial extraction kit. The rest of the tissue was stored in a refrigerator at −80°C.

### 2.6 Determination of gastric emptying rate and small intestine propulsion rate by semi-solid paste method

The whole stomach mass, empty stomach mass and semi-solid paste mass were collected and recorded, and the total length of the small intestine and the distance advanced by the semi-solid paste in the small intestine were measured and recorded and calculated according to the following formula: gastric emptying rate = [1- (whole stomach mass—empty stomach mass)/semi-solid paste mass] × 100%, small intestine propulsion rate (%) = distance of semi-solid paste advancing in small intestine/total length of small intestine × 100% (Liang et al., 2018; Zhu et al., 2020).

### 2.7 Histopathological changes in rat gastric antrum tissue observed by H&E staining

After dewaxing, the sections were stained with hematoxylin for 2 min, rinsed with running water for 5 min, stained with eosin for 90 s, rinsed with running water for 10 min, oven at 60°C for 30 min, and sealed with neutral resin to observe the histopathological changes of the gastric antrum tissue in each group.

### 2.8 Observation of mitochondrial structure of interstitial cells of cajal in rat gastric antrum by transmission electron microscope

Gastric antrum tissues were rapidly placed in 2.5% glutaraldehyde fixation for 2 h, rinsed for 10 min × 2 times, fixed with 1% osmic acid fixative for 2 h, rinsed in double-distilled water for 10 min × 2 times, dehydrated in steps of 30% → 50% → 70% ethanol for 10 min and then block-stained in 70% ethanol containing 3% dioxin acetate at 4°C, dehydrated in steps of 80% → 95% → 100% → 100% ethanol, and after 10 min × 2 times of epichlorohydrin replacement, Epon812 embedding solution was infiltrated with propylene oxide (1:1, 2 h; 2:1, overnight), and pure Epon812 embedding solution was infiltrated at 37°C for 6 h. Sections were baked at 60°C for 48 h, electronically stained with lead citrate, and observed and photographed by TEM (Hitachi, H-7650, Japan).

### 2.9 Determination of serum motilin, gastrin, superoxide dismutase, malondialdehyde content by enzyme-linked immunosorbent assay

The contents of serum MTL, GAS, SOD and MDA in rats were determined according to the instructions of the ELISA kit. The absorbance A of each sample was read at the wavelength of 450 nm on the microplate reader, and the standard curve was plotted and the concentration value of each sample was calculated according to the curve equation.

### 2.10 Determination of mitochondrial reactive oxygen species activity in rat gastric tissues by spectrophotometer

The fluorescence intensity was measured at excitation wavelength 485 nm and emission wavelength 525 nm using a spectrophotometer, following the steps of the ROS assay kit instructions.

### 2.11 Observation of ckit, light chain 3 and VADC1, dynamin-related protein 1 and translocase of the outer mitochondrial membrane 20 expression in gastric antrum tissue by immunofluorescence

After dewaxing with xylene, the antigen was retrieved with 0.01 mol/L citrate buffer for 15 min, then cooled to room temperature naturally, washed with PBS, blocked with 10% BSA at room temperature for 1 h, primary antibodies (ckit, 1:200; LC3 and VADC1, 1:200; Drp1 and Tom20, 1:200) were added overnight at 4°C. After washing with PBS, fluorescent secondary antibodies Alexa Fluor 488-labeled goat anti-rabbit IgG (H + L) and Cy3-labeled goat anti-rat IgG (H + L) (1:200) were added, respectively. Incubated for 1 h at room temperature and protected from light, washed with PBS, and nuclei stained with DAPI for 5 min. After mounting, the expressions of ckit, LC3 and VADC1, Drp1 and Tom20 were observed with a fluorescence confocal microscope.

### 2.12 Detection the expression of ckit and mitophagy-related proteins dynamin-related protein 1, Beclin1, P62, and light chain 3 in gastric antrum tissue by western blot

Mitochondria were extracted from rat gastric antrum tissue according to the instructions of the mitochondrial extraction kit, protein concentration was determined by BCA, separated by 10% SDS-PAGE electrophoresis for 90 min, transferred by wet transfer method (300 mA, 30–90 min), and closed in 5% skimmed milk for 2 h at room temperature in a shaker, rabbit anti-ckit, Drp1, LC3, Beclin1, P62, and GAPDH (1:1000) was incubated overnight at 4°C in a refrigerator shaker; the next day, the relevant secondary antibody (1:1000) was incubated for 1 h at room temperature, the membrane was washed in TBST buffer for 10 min/time × 3 times, developed in ultra-sensitive ECL luminescent solution, and the gray value of the band was analyzed by Image J.

### 2.13 Statistical analysis

SPSS 24.0 statistical software was used for analysis, and the experimental results were expressed as mean ± standard deviation (means ± SD). One-way ANOVA was used for comparison of means among multiple groups, with *p* < 0.05 representing a statistically significant difference.

## 3 Results

### 3.1 Effects of different doses of hesperidin on gastric motility in functional dyspepsia rats

#### 3.1.1 Effects of different doses of hesperidin on pathological changes of gastric antrum in functional dyspepsia rats

**FIGURE 1:**
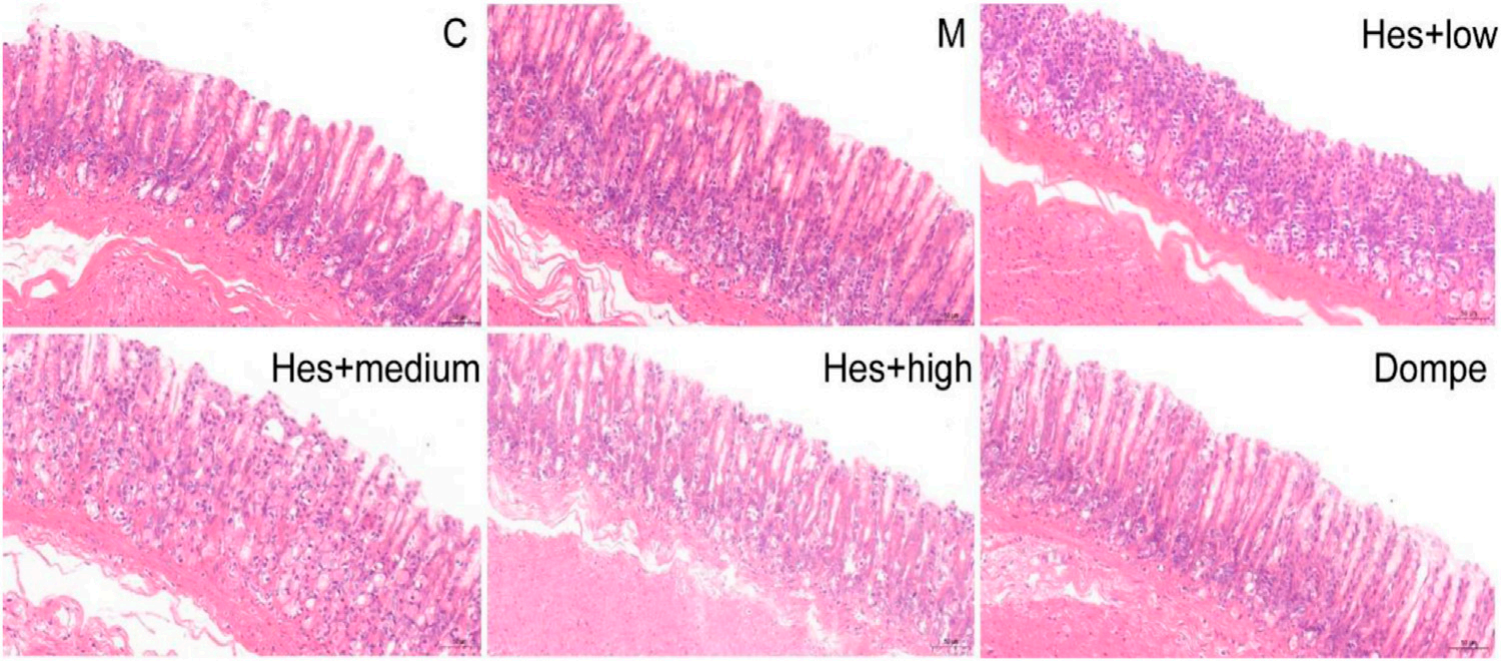
H&E staining of rat gastric antrum tissue in each group (H&E, ×200). C, control; M, model; Hes + low, Hesperidin + low; Hes + medium, Hesperidin + medium; Hes + high, Hesperidin + high; Dompe, Domperidone.

The structure of the gastric antrum was clear, the mucosa was smooth, the glandular structure was regular, and the shape of the epithelial cells and the size of the interstitium of the gastric mucosa were the same in all groups; a small amount of neutrophil infiltration was seen in the model group, the different doses of Hes group, and the Dompe group, and no pathological changes such as erosion and ulceration were seen, see Figure 1 for details.

#### 3.1.2 Effects of different doses of hesperidin on gastric emptying rate and small intestinal propulsion rate in functional dyspepsia rats

Compared with the control group, the gastric emptying rate and small intestinal propulsion rate in the model group was significantly decreased (*p* < 0.001). Compared with the model group, the gastric emptying rate and small intestinal propulsion rate in the medium-dose Hes group, high-dose Hes group and Dompe group were significantly increased (*p* < 0.05, *p* < 0.01, *p* < 0.001), as detailed in Table 1.

**TABLE 1:** Effects of different doses of hesperidin on gastric emptying rate and small intestinal propulsion rate in functional dyspepsia rats.

Group	*n*	Gastric emptying rate	Small intestinal propulsion rate
C	6	0.62 ± 0.11	0.84 ± 0.05
M	6	0.36 ± 0.09^***^	0.66 ± 0.08^***^
Hes + low	6	0.39 ± 0.05	0.69 ± 0.07
Hes + medium	6	0.48 ± 0.08^#^	0.77 ± 0.05^##^
Hes + high	6	0.49 ± 0.06^##^	0.79 ± 0.05^###^
Dompe	6	0.49 ± 0.08^#^	0.78 ± 0.05^##^

C, control; M, model; Hes + low, Hesperidin + low; Hes + medium, Hesperidin + medium; Hes + high, Hesperidin + high; Dompe, Domperidone. Data are presented as means ± SD, compared with C, ^*^ *p* < 0.05, ^**^ *p* < 0.01, ^***^ *p* < 0.001, compared with M, ^#^ *p* < 0.05, ^##^ *p* < 0.01, ^###^ *p* < 0.001.

#### 3.1.3 Effects of different doses of hesperidin on serum motilin and gastrin contents in functional dyspepsia rats

Compared with the control group, the serum MTL and GAS contents in model group were significantly decreased (*p* < 0.001); compared with the model group, the serum MTL and GAS contents in medium-dose Hes group, high-dose Hes group and Dompe group were significantly increased (*p* < 0.001), as detailed in Table 2.

**TABLE 2:** Effect of different doses of hesperidin on serum motilin (MTL) and gastrin (GAS) contents in functional dyspepsia rats.

Group	*n*	MLT	GAS
C	6	556.25 ± 9.05	505.27 ± 27.27
M	6	230.75 ± 34.84^***^	227.00 ± 22.59^***^
Hes + low	6	255.02 ± 44.33	262.22 ± 31.88
Hes + medium	6	399.28 ± 14.52^###^	387.10 ± 53.15^###^
Hes + high	6	345.97 ± 11.71^###^	338.98 ± 7.40^###^
Dompe	6	466.42 ± 13.62^###^	422.12 ± 53.25^###^

C, control; M, model; Hes + low, Hesperidin + low; Hes + medium, Hesperidin + medium; Hes + high, Hesperidin + high; Dompe, Domperidone. MLT&GAS, pg/ml. Data are presented as means ± SD, compared with C, ^*^ *p* < 0.05, ^**^ *p* < 0.01, ^***^ *p* < 0.001, compared with M, ^#^ *p* < 0.05, ^##^ *p* < 0.01, ^###^ *p* < 0.001.

#### 3.1.4 Effects of different doses of hesperidin on the expression of ckit in the gastric antrum tissue of functional dyspepsia rats

**FIGURE 2:**
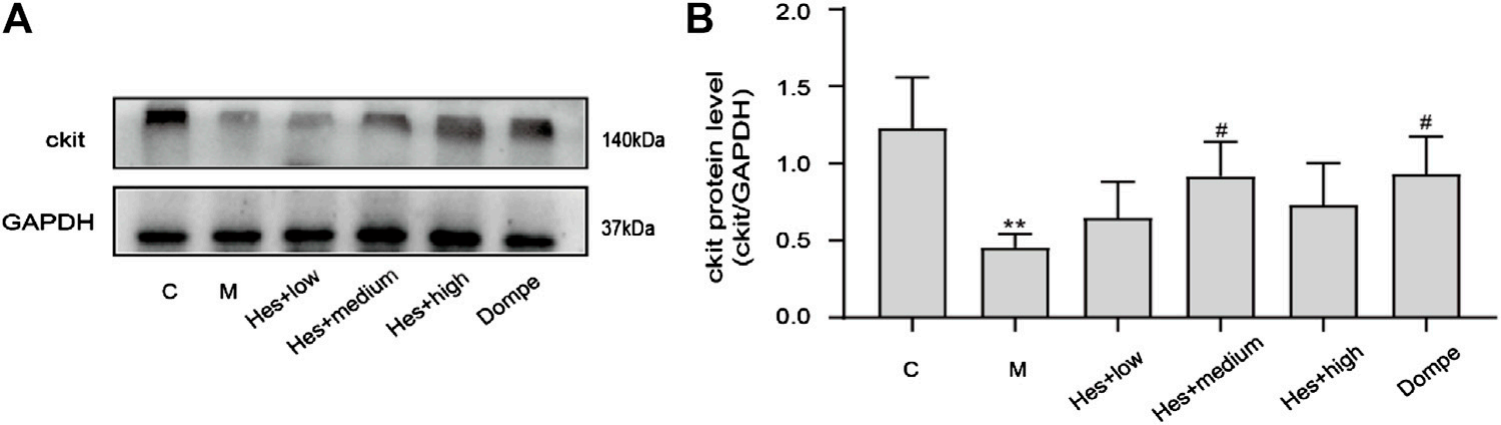
Expression of ckit in functional dyspepsia rats detected by Western blot. **(A)** Western blot. **(B)** Results of ckit Western blot. C, control; M, model; Hes + low, Hesperidin + low; Hes + medium, Hesperidin + medium; Hes + high, Hesperidin + high; Dompe, Domperidone. Data are presented as means ± SD, compared with C, *p < 0.05, **p < 0.01, compared with M, ^#^ *p* < 0.05.

Compared with the control group, the expression of ckit in the model group was significantly decreased (*p* < 0.01); in comparison with the model group, which was increased in the middle-dose Hes group and Dompe group (*p* < 0.05), see Figure 2 for details.

### 3.2 Effect of hesperidin on mitochondrial damage and mitophagy of interstitial cells of cajal in functional dyspepsia rats under mdivi-1 intervention

#### 3.2.1 Effect of hesperidin on serum malondialdehyde and superoxide dismutase content in functional dyspepsia rats

**FIGURE 3:**
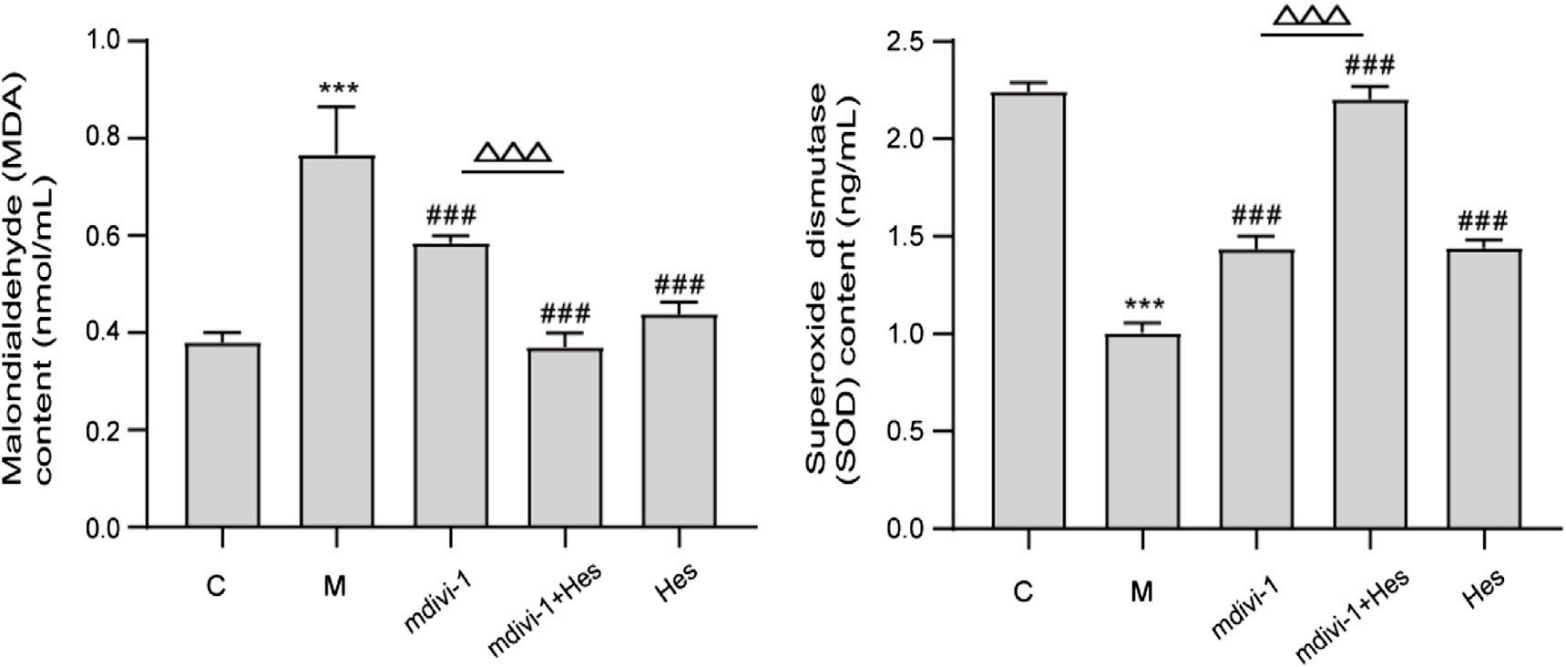
Effect of hesperidin on serum malondialdehyde (MDA) and superoxide dismutase (SOD) contents in functional dyspepsia rats. C, control; M, model; mdivi-1+Hes, mdivi-1+Hesperidin; Hes, Hesperidin; MDA, nmol/ml; SOD, ng/ml; Data are presented as means ± SD, compared with C, *p < 0.05, **p < 0.01, ***p < 0.001, compared with M, ^#^ *p* < 0.05*,* ^##^ *p* < 0.01, ^###^ *p* < 0.001, compared with mdivi-1, ^△^ *p* < 0.05, ^△△^ *p* < 0.01,^△△△^ *p* < 0.001.

Compared with the control group, the MDA content in the model group was significantly increased (*p* < 0.001), whereas SOD content was significantly decreased (*p* < 0.001); compared with the model group, the MDA content of mdivi-1 group, mdivi-1+Hes group and Hes group were significantly decreased (*p* < 0.001) and SOD content was significantly increased (*p* < 0.001); while compared with the mdivi-1 group, the mdivi-1+Hes group had lower MDA content (*p* < 0.001) and higher SOD content (*p* < 0.001), as detailed in Figure 3.

#### 3.2.2 Effect of hesperidin on mitochondrial reactive oxygen species activity in functional dyspepsia rats

**FIGURE 4:**
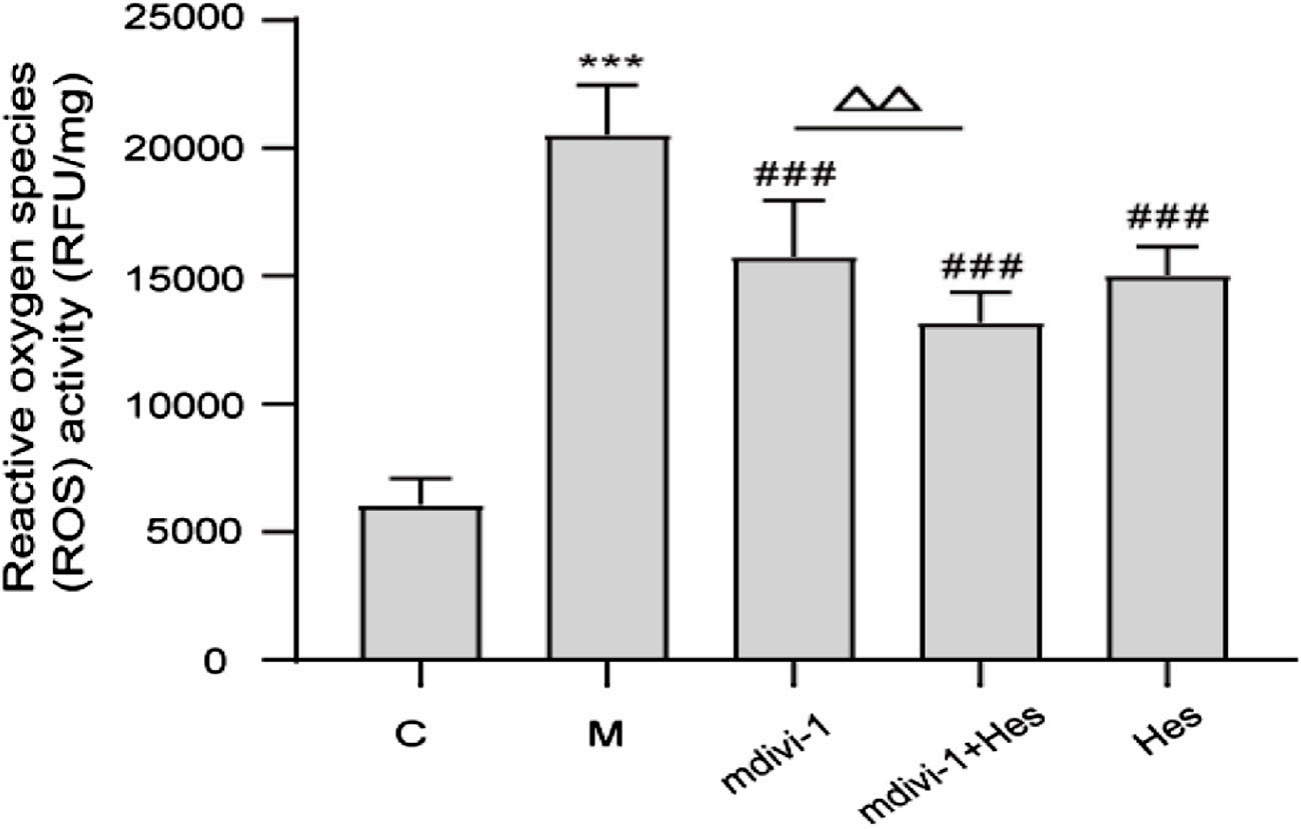
Effect of hesperidin on mitochondrial reactive oxygen species (ROS) activity in functional dyspepsia rats. C, control; M, model; mdivi-1+Hes, mdivi-1+Hesperidin; Hes, Hesperidin; ROS, RFU/mg; Data are presented as means ± SD, compared with C, *p < 0.05, **p < 0.01, ***p < 0.001, compared with M, ^#^ *p* < 0.05*,* ^##^ *p* < 0.01, ^###^ *p* < 0.001, compared with mdivi-1, ^△^ *p* < 0.05, ^△△^ *p* < 0.01.

Compared with the control group, the activity of mitochondrial ROS in the model group was significantly increased (*p* < 0.001); compared with the model group, the activity of mitochondrial ROS in the mdivi-1 group, mdivi-1+Hes group and Hes group was significantly decreased (*p* < 0.001); and compared with the mdivi-1 group, mdivi-1+Hes group had lower mitochondrial ROS activity (*p* < 0.01), see Figure 4 for details.

#### 3.2.3 Effect of hesperidin on the ultrastructure of the interstitial cells of cajal mitochondria in gastric antrum tissue of functional dyspepsia rats

**FIGURE 5:**
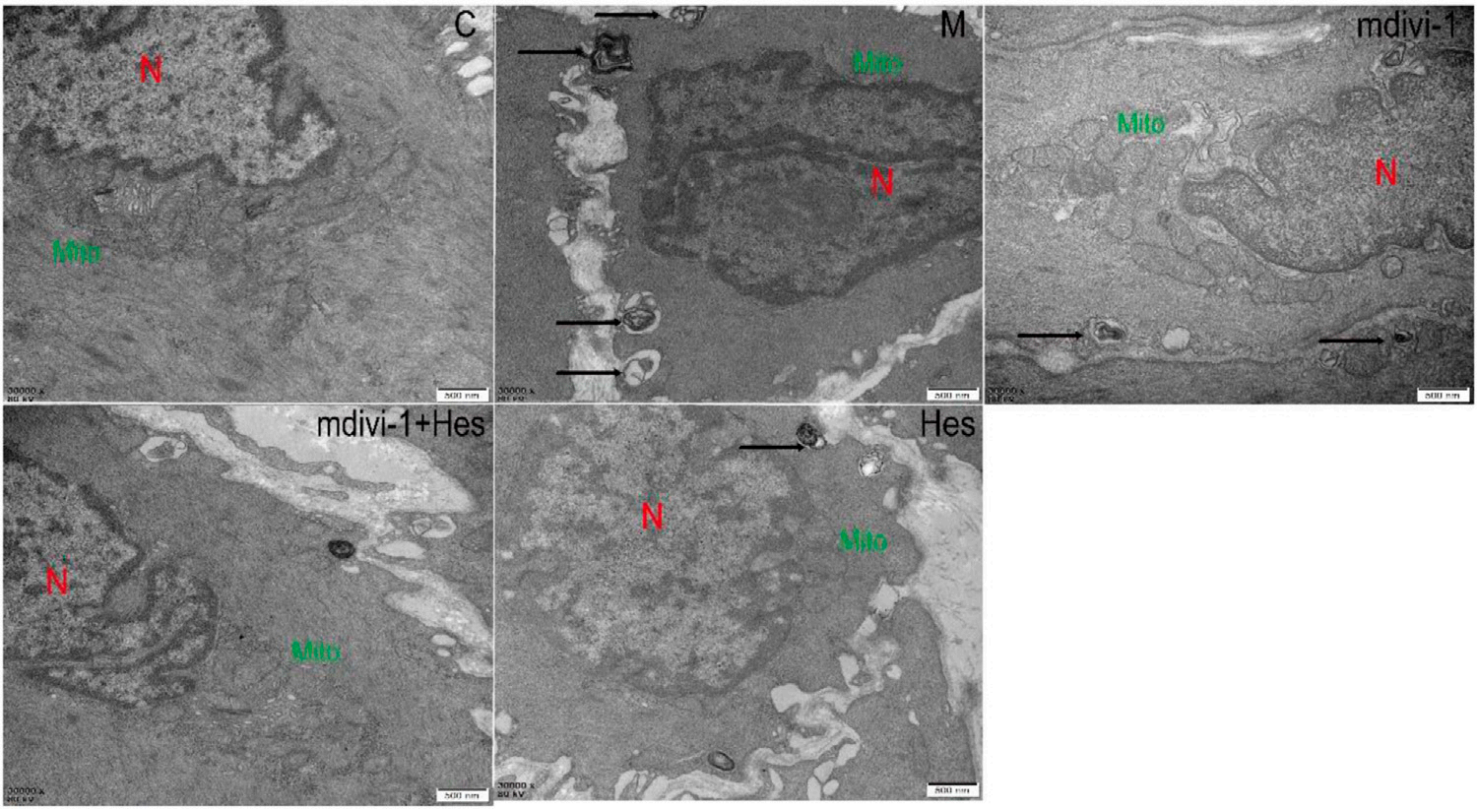
Effects of hesperidin on the ultrastructure of the interstitial cells of cajal (ICC) mitochondria in gastric antrum tissue of functional dyspepsia rats (TEM, ×30000). C, control; M, model; mdivi-1+Hes, mdivi-1+Hesperidin; Hes, Hesperidin. Scale bar: 500 nm, N stands for nucleus, Mito for mitochondria, and autolysosomes are marked with arrows.

In the control group, the rats had a clear morphological structure of ICC with long shuttle shape or oval shape, intact nuclear membrane, high cristae density and large number of mitochondria in the cytoplasm. In the model group, the mitochondrial morphology was blurred, the number of mitochondria was reduced, the mitochondria were swollen and dilated, vacuolated lesions were present, and a large number of autophagic lysosomes were visible. The mdivi-1 group, mdivi-1+Hes group and Hes group were more structurally intact, with long shuttle-shaped mitochondria, more intact nuclear membranes, higher cristae density and a small number of mitochondria splitting and fusing, among which, a small number of autophagic lysosomes were visible in the mdivi-1 group and Hes group, as detailed in Figure 5.

#### 3.2.4 The effect of hesperidin on the expression of ckit in gastric antrum of functional dyspepsia rats

**FIGURE 6:**
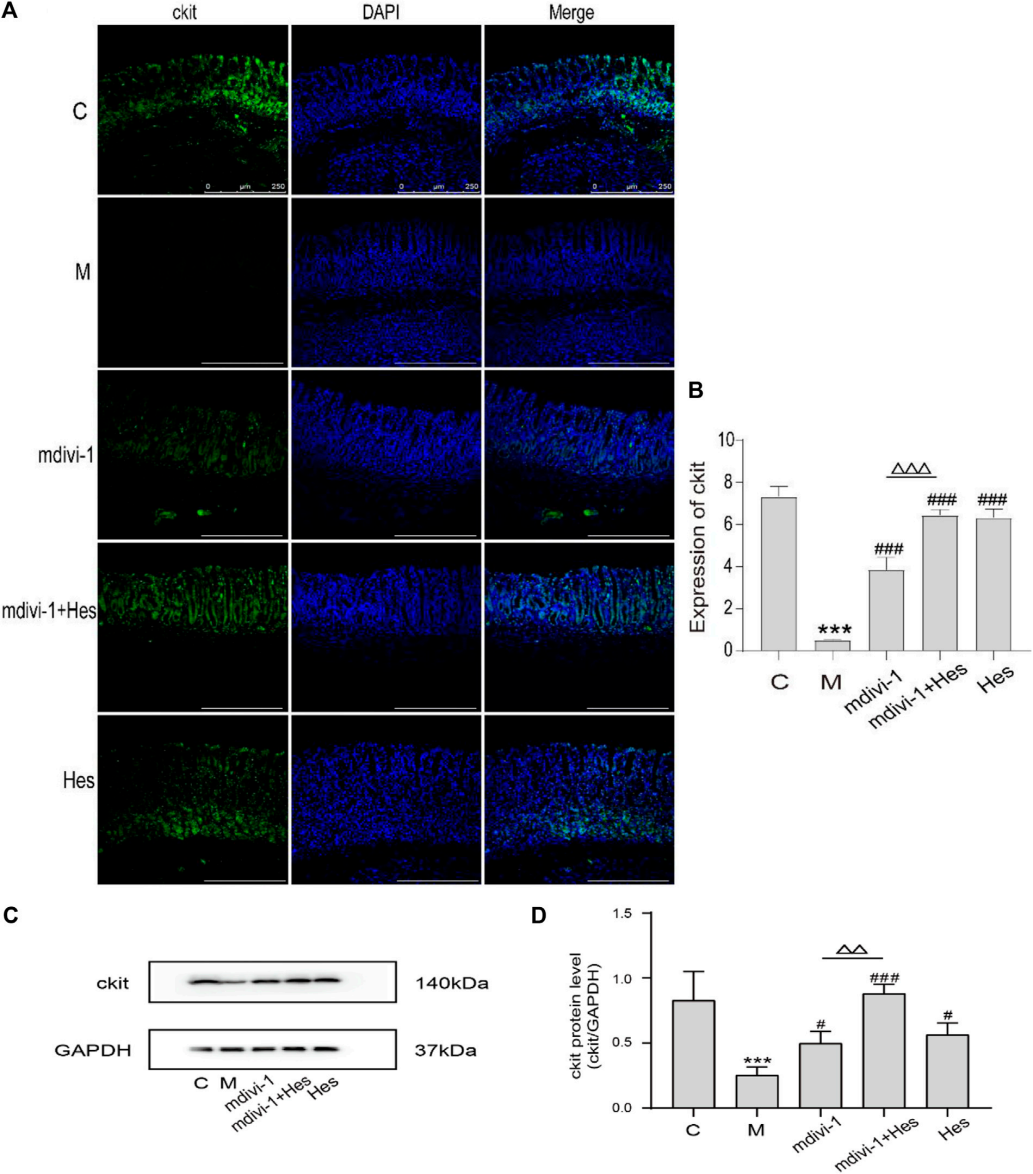
Expression of ckit in functional dyspepsia rats detected by Immunofluorescence and Western blot. **(A)** Immunofluorescence, Scale bar: 250 μm. **(B)** Results of ckit Immunofluorescence. **(C)** Western blot. **(D)** Results of ckit Western blot. C, control; M, model; mdivi-1+Hes, mdivi-1+Hesperidin; Hes, Hesperidin; Data are presented as means ± SD, compared with C, *p < 0.05, **p < 0.01, ***p < 0.001, compared with M, ^#^ *p* < 0.05, ^##^ *p* < 0.01, ^###^ *p* < 0.001, compared with mdivi-1, ^△^ *p* < 0.05, ^△△^ *p* < 0.01, ^△△△^ *p* < 0.001.

The fluorescence results showed that compared with the control group, the expression of ckit in the model group was significantly decreased (*p* < 0.001); compared with the model group, the expression of ckit in the mdivi-1 group, mdivi-1+Hes group and Hes group was significantly increased (*p* < 0.001); and the expression of ckit was higher in the mdivi-1+Hes group compared with the mdivi-1 group (*p* < 0.001); see Figures 6A,B for details. WB results suggested that compared with the control group, the expression of ckit in the model group was significantly decreased (*p* < 0.001); compared with the model group, the expression of ckit in the mdivi-1 group, mdivi-1+Hes group and Hes group was increased (*p* < 0.05, *p* < 0.001); and the expression of ckit was higher in the mdivi-1+Hes group compared to the mdivi-1 group (*p* < 0.01), as detailed in Figures 6C,D.

#### 3.2.5 Effect of hesperidin on fluorescence co-localization of dynamin-related protein 1 and translocase of the outer mitochondrial membrane 20, microtubule associated protein 1 light chain 3 and voltage dependent anion channel 1 in gastric antrum of functional dyspepsia rats

**FIGURE 7:**
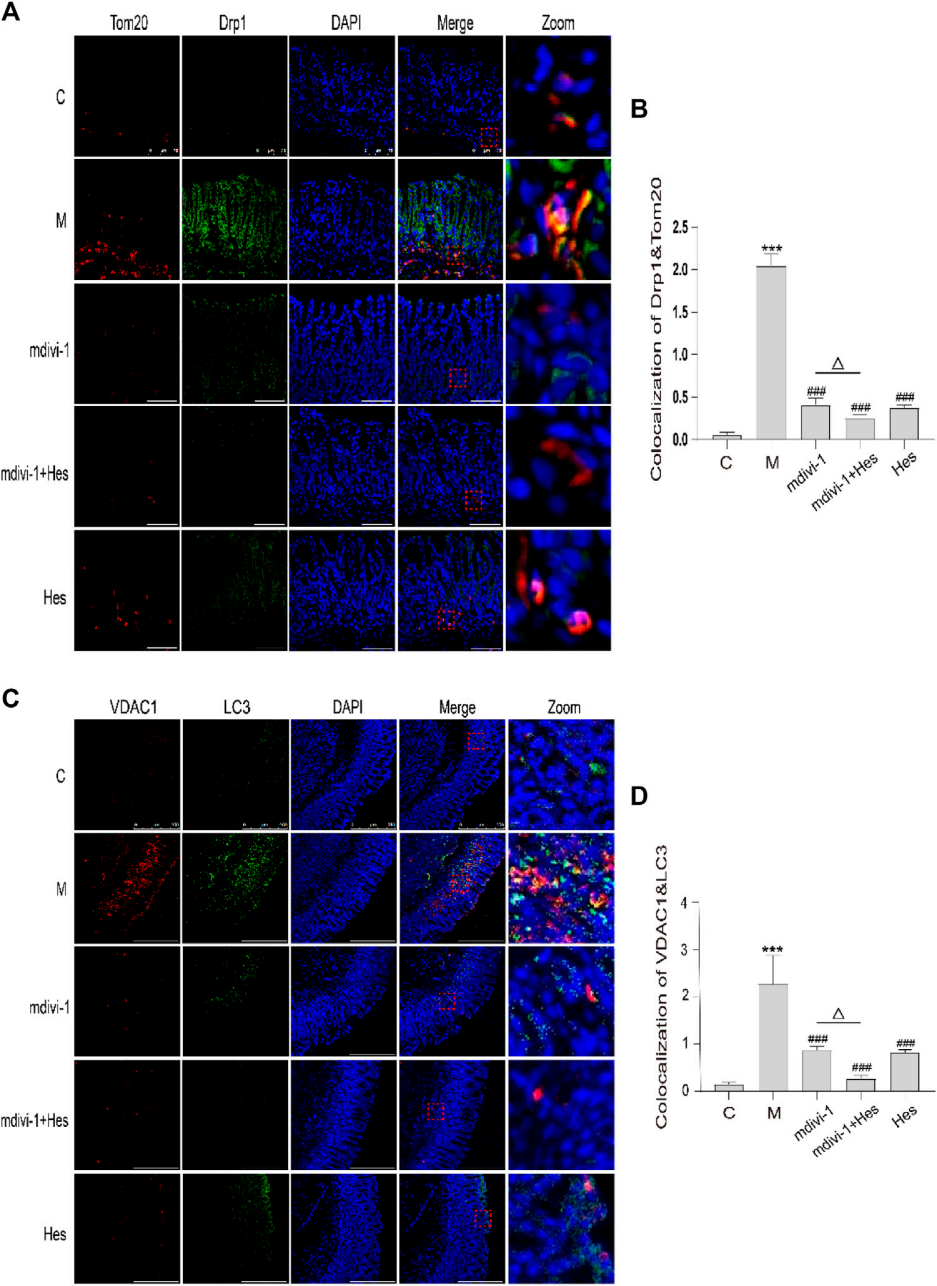
Effects of hesperidin on the fluorescence co-localization expression of dynamin-related protein 1 (Drp1) and translocase of the outer mitochondrial membrane 20 (Tom20), microtubule associated protein 1 light chain 3 (LC3) and voltage dependent anion channel 1 (VDAC1) in gastric antrum of functional dyspepsia rats. **(A)** Immunofluorescence of Drp1 and Tom20, Scale bar: 75 μm. **(B)** Colocalization of Drp1 and Tom20 Immunofluorescence. **(C)** Immunofluorescence of LC3 and VDAC1, Scale bar: 250 μm **(D)** Colocalization of LC3 and VDAC1 Immunofluorescence. C, control; M, model; mdivi-1+Hes, mdivi-1+Hesperidin; Hes, Hesperidin. Data are presented as means ± SD, compared with C, *p < 0.05, **p < 0.01, ***p < 0.001, compared with M, ^#^ *p* < 0.05*,* ^##^ *p* < 0.01, ^###^ *p* < 0.001, compared with mdivi-1, ^△^ *p* < 0.05.

Compared with the control group, the co-localization expression of Drp1 and Tom20 was significantly increased in the model group (*p* < 0.001); compared with the model group, the co-localization expression was significantly decreased in the mdivi-1, mdivi-1+Hes and Hes groups (*p* < 0.001); and the co-localization expression was less in the mdivi-1+Hes group compared with the mdivi-1 group (*p* < 0.05), see Figures 7A,B for details. Compared with the control group, the co-localization expression of LC3 and VDAC1 was increased in the model group (*p* < 0.001); LC3 and VDAC1 co-localization expression was decreased in the mdivi-1, mdivi-1+Hes and Hes groups compared with the model group (*p* < 0.001); and less co-localization expression was observed in the mdivi-1+Hes group compared to the mdivi-1 group (*p* < 0.05), as detailed in Figures 7C,D.

#### 3.2.6 Effects of hesperidin on the protein expression of mitochondrial dynamin-related protein 1, P62, Beclin1, and light chain 3 in functional dyspepsia rats

**FIGURE 8:**
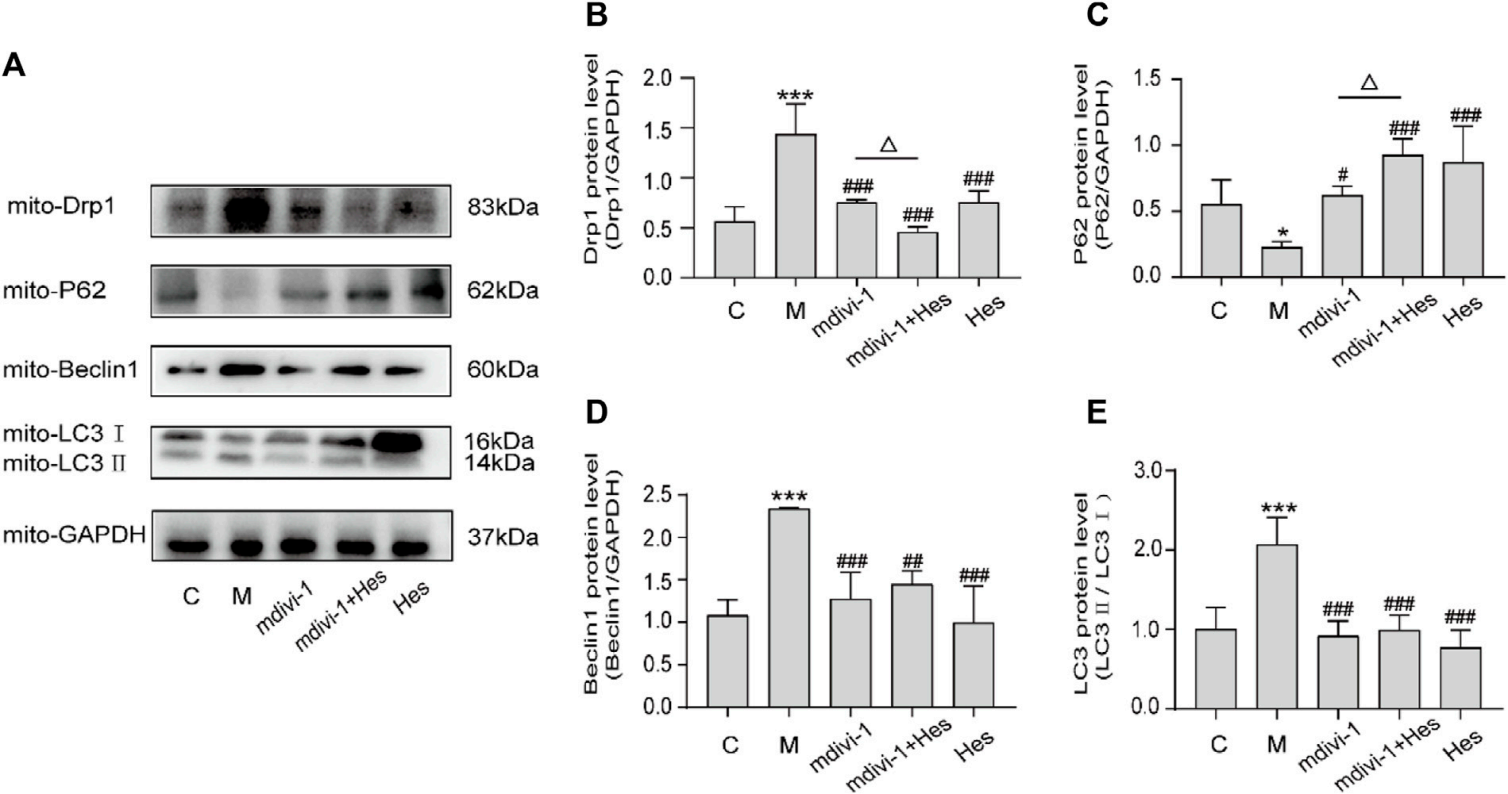
Effects of hesperidin on the protein expression of mitochondrial Drp1, P62, Beclin1, and LC3 in functional dyspepsia rats. **(A)** Western blot. **(B–E)** Expression of Drp1, P62, Beclin1 and LC3. C, control; M, model; mdivi-1+Hes, mdivi-1+Hesperidin; Hes, Hesperidin. Data are presented as means ± SD, compared with C, *p < 0.05, **p < 0.01, ***p < 0.001, compared with M, ^#^ *p* < 0.05, ^##^ *p* < 0.01, ^###^ *p* < 0.001, compared with mdivi-1, ^△^ *p* < 0.05.

Compared with the control group, the protein expressions of mitochondrial Drp1, Beclin1, and LC3 in the model group were significantly increased (*p* < 0.001), and the protein expression of P62 was decreased (*p* < 0.05); compared with the model group, the protein expressions of Drp1, Beclin1, LC3 in the mdivi-1, mdivi-1+Hes and Hes groups were decreased significantly (*p* < 0.01, *p* < 0.001) and the protein expression of P62 was significantly higher (*p* < 0.05, *p* < 0.001); and compared with the mdivi-1 group, the mdivi-1+Hes group had lower Drp1 expression (*p* < 0.05) and higher P62 protein expression (*p* < 0.05), as detailed in Figure 8.

## 4 Discussion

The incidence of functional dyspepsia (FD) accounts for approximately 20–40% of gastroenterology outpatient visits and seriously affects the quality of life of patients (Ohara et al., 2011; Jin and Son, 2018). Related studies have shown that gastrointestinal hormones such as motilin (MTL) and gastrin (GAS) play an important role in the occurrence and development of FD (Zhao et al., 2015; Deng et al., 2018). MTL can induce enhanced smooth muscle movement of the gastrointestinal tract, promote gastrointestinal peristalsis, and accelerate gastric emptying; GAS acts on almost the entire gastrointestinal tract, promoting contraction of the gastric antrum and gastric body, increasing GAS acts on almost the entire gastrointestinal tract, promotes the contraction of the gastric antrum and gastric body, increases the movement of the gastrointestinal tract, and also promotes the proliferation of the epithelium of the gastrointestinal mucosa. Domperidone improves postprandial epigastric distension, epigastric pain, belching and early satiety in FD patients, and therefore serves as a positive control drug (Shokry-Shirvani et al., 2012). In this study, H&E staining indicated that no significant organic changes were seen in the gastric antrum tissue of rats in each group, and the gastric emptying rate and small intestine propulsion rate of rats in the model group were significantly lower than those in the control group, which was consistent with the disease characteristics of FD and suggested successful modeling. Compared with the control group, the serum MTL and GAS contents decreased, and ckit protein expression decreased in the model group; after hesperidin intervention, the serum MTL and GAS levels increased, and ckit protein expression increased. Therefore, hesperidin can effectively promote gastric motility in FD rats, and the medium-dose hesperidin was selected for follow-up experiments.

As the main site of reactive oxygen species (ROS) production in cells, mitochondria are responsible for intracellular redox signaling regulation and maintenance of oxidative homeostasis through a range of antioxidants (Zorov et al., 2014). Oxidative stress can increase ROS and decrease the formation of antioxidant defenses. The reduction in the activity of antioxidant enzymes such as superoxide dismutase (SOD) promotes oxidative attack on cells. Once the redox balance is disrupted, SOD reacts with oxygen molecules and scavenges superoxide anions that are harmful to the organism thus achieving antioxidant stress (Liu et al., 2020; Dokumacioglu et al., 2021). Malondialdehyde (MDA) is the carbonyl group produced during lipid peroxidation and is widely used to determine oxidative stress. SOD activity decreases during oxidative stress and produces excess lipid peroxidation product MDA, which can indirectly reflect the degree of mitochondrial peroxidative damage (Tian et al., 2019; Dokumacioglu et al., 2021). The results of this study showed that compared with the control group, the serum MDA content of the model group was increased, the SOD content was decreased, and the mitochondrial ROS level was increased. Transmission electron microscopy (TEM) showed a decrease in the number of mitochondria, swollen and dilated morphology, and even vacuoles in the model group, indicating oxidative stress and mitochondrial damage in the ICC mitochondria of FD rats. Previous studies have further shown that after mdivi-1 intervention, dynamin-related protein 1 (DRP1) is activated by GTPase hydrolysis and oligomerized around the outer mitochondrial outer membrane, which triggers fission and mitochondrial fracture (Rovira-Llopis et al., 2017; Fonseca et al., 2019). The delicate balance between mitochondrial fission and fusion events is disturbed by elevated Drp1 levels, which promotes mitochondrial fission and ultimately impairs mitochondrial function, leading to increased ROS production (Kim et al., 2016; Roy and Sesaki, 2021). If Drp1 protein expression was increased in human umbilical vein endothelial cells under high glucose conditions, increased mitochondrial ROS production and decreased SOD activity induced further mitochondrial fission and apoptosis (Hao et al., 2019). The results of this study suggested that mdivi-1 intervention reduced mitochondrial ROS and serum MDA levels and increased serum SOD content in gastric antrum tissue compared with the model group, and the morphological structure of ICC mitochondria was clearer and the nuclear membrane was more intact under TEM, suggesting that mdivi-1 intervention could reduce mitochondrial oxidative stress in FD rats. The results of the hesperidin group were similar to those of the mdivi-1 group. However, the mdivi-1+hesperidin group had the lowest ROS and MDA, the highest SOD content, and better morphological structure of ICC mitochondria, suggesting that hesperidin could further reduce mitochondrial oxidative stress and improve mitochondrial damage in FD rats under mdivi-1 intervention.

Mitophagy is a normal physiological activity of cells and is one of the forms of cellular autophagy. Mitophagy refers to the damage that occurs when mitochondria are depolarized in response to external stimuli such as oxidative damage/nutrient deficiency/cellular senescence, and the damaged mitochondria are characteristically encapsulated into autophagosomes and fused with lysosomes, thus completing the degradation of the damaged mitochondria and maintaining intracellular environmental homeostasis. Mitophagy involves three main stages: mitochondrial division, autophagosome assembly and fusion with lysosomes to degrade the damaged mitochondria (Patergnani and Pinton, 2015; Springer and Macleod, 2016; Ma et al., 2020). TEM suggested that a large number of autophagic lysosomes were visible in ICC cells of the model group compared with the control group; compared with the model group, only a few autophagic lysosomes were visible in the mdivi-1 and hesperidin groups, and no autophagic lysosomes were seen in the mdivi-1+hesperidin group. To further confirm whether the mitochondria were wrapped by autophagosomes, we co-localized the expression of Drp1, a key protein regulating mitophagy, with the mitochondrial marker Tom20; the mitochondrial outer membrane protein VDAC1 and the autophagosomal marker protein LC3 by Immunofluorescence. Compared with the control group, the co-localized expression of Drp1 and Tom20, LC3 and VDAC1 in the model group increased; while the co-localized expression of those in the mdivi-1 group, mdivi-1+hesperidin group and hesperidin group decreased, indicating that mdivi-1 acts as an inhibitor of mitochondrial splitting proteins and reduces mitophagy, and the mitophagy was also significantly improved in the hesperidin group, with the best synergistic effect of hesperidin and mdivi-1, thus revealing that hesperidin could further improve mitophagy in ICC of FD rats under the intervention of mdivi-1.

Drp1 can interact with LC3 and thus induce mitophagy (Wu et al., 2016). LC3 is a marker of mitophagy, and when mitophagy occurs, LC3Ⅰ is modified by ubiquitination and binds to phosphatidylethanolamine on the surface of autophagic vesicles to form LC3Ⅱ, which is localized on the surface of autophagosomal membranes, and LC3Ⅱ and LC3Ⅱ/LC3Ⅰ represent to some extent the level of mitochondrial autophagy in the body (Chifenti et al., 2013). P62, a specific substrate for mitophagy, directly binds LC3 and is selectively degraded by autophagy, negatively regulating mitochondrial autophagic activity (Sulkshane et al., 2021). Beclin1 promotes the conversion of LC3Ⅰ to LC3Ⅱ and accelerates the fusion of autophagosomes with lysosomes, activating mitophagy, and is a key factor in evaluating mitochondrial autophagic activity (Jakhar et al., 2016). The results revealed that the protein expressions of mitochondrial Drp1, LC3, and Beclin-1 were down-regulated and the expression of P62 protein was increased after mdivi-1 or hesperidin intervention compared with the model group; the expression of ckit in gastric antrum tissues was increased. Mdivi-1, as an inhibitor of mitochondrial splitting proteins, was seen to reduce mitochondrial autophagy and thus exert a pro-gastric motility effect. In the mdivi-1+hesperidin group, the expression of mitochondrial Drp1 protein was lower and the expression of P62 protein was higher; the expression of ckit protein in the gastric antrum tissues was further up-regulated. The above results indicated that mdivi-1 synergistically regulated Drp1-mediated mitochondrial autophagy with hesperidin.

In conclusion, we believe that hesperidin can enhance mitochondrial activity, inhibit ICC mitophagy, and promote gastric motility in FD rats through the Drp1 signaling pathway, thus effectively preventing and treating FD.

## Data Availability

The raw data supporting the conclusion of this article will be made available by the authors, without undue reservation.
